# Assessing Usage and Usability of a Narrative-Based Psychoeducational Digital Intervention to Improve Medication Adherence Among Individuals With Schizophrenia in a Stable Phase: Mixed Methods Study

**DOI:** 10.2196/59175

**Published:** 2026-01-20

**Authors:** Dian Zhu, Fangyuan Chang, Hongyi Yang, Yiwen Wei, Zhao Liu

**Affiliations:** 1 School of Art Design and Media, East China University of Science and Technology, Shanghai, China; 2 School of Design, Shanghai Jiao Tong University, No.800, Dongchuan RD, Shanghai, 200240, China, +86-34205152

**Keywords:** schizophrenia, psychoeducation, medication adherence, cognitive behavior therapy, mixed methods study

## Abstract

**Background:**

Nonadherence to antipsychotic medication remains one of the most substantial challenges in the management of schizophrenia, contributing to relapse, rehospitalization, and functional decline. Although psychoeducational interventions are a key intervention for relapse prevention, traditional formats often lack interactivity and cultural resonance, thereby limiting engagement and sustained impact. Digital health innovations offer an opportunity to improve both treatment adherence and user experience, but evidence in schizophrenia populations remains limited.

**Objective:**

This study aimed to evaluate the usage patterns, usability, and effectiveness of a narrative-based psychoeducational digital intervention designed to enhance medication adherence among individuals with schizophrenia in the maintenance phase. By employing a mixed methods design, the study integrated quantitative measures of adherence and functioning with qualitative insights into participants’ experiences and perceptions.

**Methods:**

A 6-month parallel mixed methods randomized controlled trial was conducted in community mental health settings in Shanghai. Seventy individuals with schizophrenia in a stable phase were randomly assigned (1:1) to the intervention group, which received the digital narrative-based psychoeducation application (Healing Town) in addition to routine community care, or to the control group, which received routine community rehabilitation only. Quantitative evaluation focused on medication adherence, drug attitude, social functioning, and psychiatric symptoms. In parallel, qualitative data were collected through semistructured interviews with patients, caregivers, and clinicians to examine intervention usage, usability, engagement, and perceived impact.

**Results:**

Seventy participants (mean age 44.2, SD 8.057 y; 61% male) were enrolled, and 69 (98.6%) completed the 6-month trial, with one dropout during the intervention period. At 6 months, the intervention group showed significantly higher medication adherence (mean difference 1.27, 95% CI 0.30‐2.24; *P*=.02) and more positive drug attitudes (mean difference 3.41, 95% CI 1.18‐5.65; *P*=.002) compared with controls. Improvements in social functioning were significant within the intervention group (*P*=.03) but not between groups. No significant group differences were observed in psychiatric symptoms. Qualitative findings identified three overarching themes: (1) adherence and usability—patients reported enhanced treatment knowledge, confidence, and motivation, though some described challenges with feedback tone and pacing; (2) experiences and attitudes—users valued cultural relevance, immersive narratives, and gamified elements but noted occasional overstimulation; and (3) expectations and recommendations—participants expressed demand for personalized features, reminders, and dynamic content to sustain engagement.

**Conclusions:**

This mixed methods study provides preliminary evidence that a narrative-based digital psychoeducational intervention may enhance medication adherence and attitudes toward medication among individuals with schizophrenia in the maintenance phase, while being perceived as engaging, usable, and culturally relevant. Furthermore, the qualitative findings suggest that supportive feedback, adaptive difficulty, and personalized features may enhance user motivation and optimize future scalability. Overall, this narrative-based digital psychoeducation represents a promising and potentially cost-effective approach to supporting community-based psychiatric rehabilitation, meriting further longitudinal and multisite investigation.

## Introduction

Schizophrenia is a chronic and severe mental disorder characterized by pervasive impairments in cognition, emotion, perception, and social functioning. Long-term use of antipsychotic medication is typically required to manage symptoms, prevent relapse, and reduce the risk of hospitalization [1-3]. However, despite advances in pharmacological treatments, approximately 50% of outpatients experience suboptimal medication adherence, which contributes to symptom exacerbation, frequent hospitalizations, and an elevated risk of suicide [4-6]. Improving adherence is not only critical for therapeutic success but also essential for enhancing long-term functional outcomes and quality of life [7].

Patients’ subjective understanding of their illness and treatment plays a pivotal role in medication-taking behavior. Misconceptions about drug efficacy or side effects, limited coping strategies, and insufficient illness insight may all diminish motivation to adhere to prescribed regimens. Psychoeducation, a core intervention aimed at enhancing illness awareness and promoting adherence, has been widely implemented in schizophrenia care. It has demonstrated effectiveness in supporting symptom recognition, self-management, and functional recovery. Nevertheless, traditional psychoeducation programs often rely heavily on didactic instruction and lack interactivity, making them less suited for individuals who may experience impairments in attention, memory, and metacognition [8]. Moreover, impaired insight—often associated with prefrontal dysfunction—can lead to resistance toward biomedical explanations, especially when these conflict with patients’ personal experiences or cultural beliefs [9,10].

In recent years, digital psychoeducation has shown great promise in enhancing treatment adherence among individuals with mental health conditions. Its advantages lie in offering highly interactive, contextualized, and personalized feedback. By integrating cognitive-behavioral strategies, gamified elements, and multimedia delivery, digital interventions have improved the accessibility of health information while enhancing user engagement and behavioral follow-through [11-13]. A particularly promising development is the emergence of narrative-based digital psychoeducation, which has demonstrated unique potential in reshaping health beliefs, eliciting emotional resonance, and strengthening treatment motivation [14]. Rooted in narrative psychology, this approach emphasizes that when individuals engage emotionally and cognitively in realistic, emotionally rich scenarios and characters, their beliefs about health, attitudes toward illness, and behavioral intentions can be reconstructed through simulated experience and observational learning [15]. Narrative interventions have shown encouraging outcomes in other areas of health education, particularly in sustaining behavior change over time [16].

Although some recent interventions targeting individuals with schizophrenia have begun to incorporate immersive, graphical narratives to enhance engagement and comprehension, this area of research remains in its early stages [17]. As Keats once suggested, narrative analysis should integrate spoken, written, and visual modalities to enable multidimensional expressions of complex psychological experiences [18]. However, many existing digital interventions still emphasize information delivery and sensory presentation, with limited attention to how patients construct meaning, express themselves, or reshape their attitudes toward treatment through narrative. This lack of integration between narrative structures and individual psychological mechanisms may undermine the effectiveness of such interventions in fostering motivation and medication adherence. In the context of long-term schizophrenia management, medication adherence is not only a key predictor of relapse and prognosis but also a reflection of the individual’s cognitive, emotional, and attitudinal engagement with treatment. Therefore, conventional educational strategies alone may fall short in activating patients’ intrinsic motivation and participatory engagement.

To address this gap, we developed an interactive, narrative-based digital application and conducted a 6-month randomized controlled trial (RCT) combined with qualitative interviews. Using a mixed methods design, we integrated quantitative and qualitative findings to evaluate the usability and user experience of the narrative-based digital psychoeducational application among individuals with schizophrenia in the maintenance phase. In addition, we examined how these user-centered outcomes were related to key 6-month end points, including medication adherence, social functioning, and clinical symptoms.

## Methods

### Study Design

A mixed methods approach was adopted in this study [19,20] following the ethical guidelines for human research outlined by the American Psychological Association [21].

In January 2023, five experienced psychiatrists contributed to the story script design, while three individuals with schizophrenia evaluated the visual style. After finalizing the script and visuals, the project moved to the application development phase.

**Figure 1.**
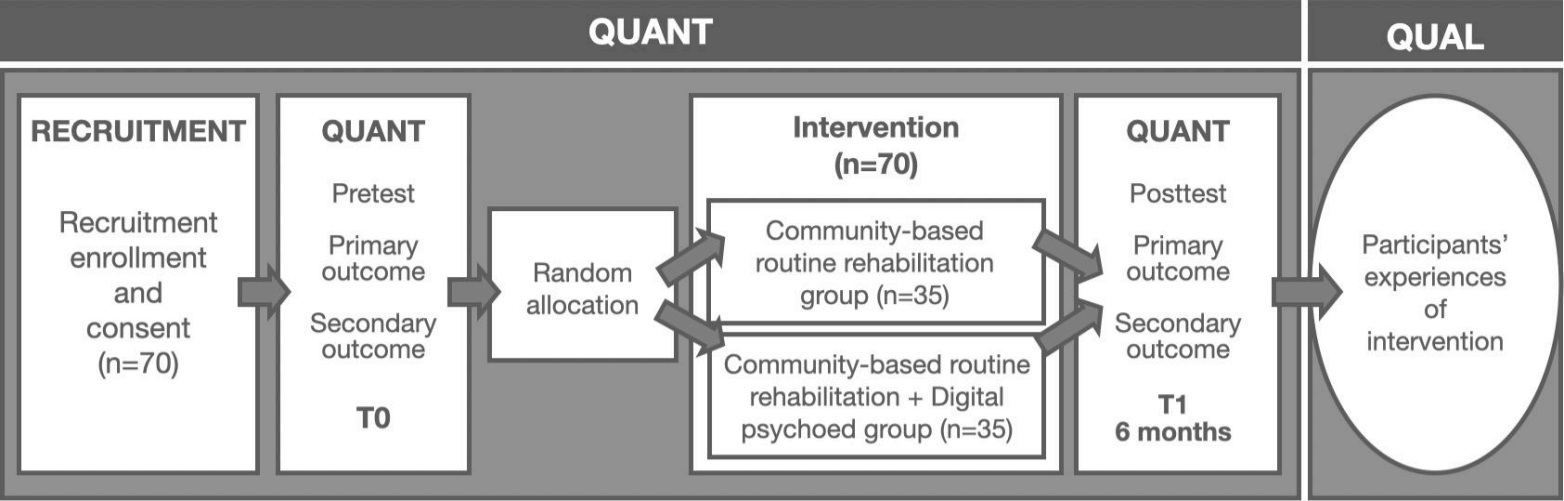
Study design of the mixed methods study to evaluate a narrative-based psychoeducational digital intervention. QUAL: qualitative; QUANT: quantitative.

Participants were recruited after providing written informed consent and screened for eligibility. Potential participants were identified through the Shanghai Mental Health Information Management System. Community psychiatrists screened eligible individuals and provided study information during routine follow-up visits. Those who expressed interest were subsequently contacted by research staff, who obtained written informed consent. The primary intervention phase was conducted between March 2024 and October 2024, lasting 6 months in total. During this period, participants attended five supervised in-person sessions each week, in which they completed the narrative-based psychoeducation program under therapist guidance (Figure 1). Baseline and postintervention assessments were conducted. At baseline, participants completed demographic surveys and medication adherence scales. All participants were trained to use the application developed by the research team. The same assessments were repeated at subsequent time points. After the intervention, structured interviews were conducted using standardized procedures. These included think-aloud protocols and semistructured interviews, with responses recorded and later digitized. Audio recordings were made for further analysis.

The quantitative component of this study was derived from a previously conducted parallel, two-arm RCT in Shanghai, implemented in accordance with the CONSORT (Consolidated Standards of Reporting Trials; Checklist 1) guidelines [22]. Participants were randomly assigned in a 1:1 ratio to either a community-based usual care group or a digital psychoeducational intervention group, with a 6-month intervention period. The usual care group received standard public health services, including regular home visits, participation in community mental health center rehabilitation programs, and education on maintaining medication adherence. In addition to these services, the intervention group participated in 5 weekly sessions of the narrative-based digital psychoeducation, each lasting 15 to 20 minutes. Each intervention site was staffed with one to two mental health professionals whose role was limited to resolving technical issues with the devices.

Following the 6-month quantitative assessment, a qualitative study was conducted in the intervention group. Semistructured interviews were carried out with all 35 users of the narrative-based digital psychoeducational intervention and 5 mental health professionals (with expertise spanning psychology, public health management, and nursing), ensuring that thematic saturation was achieved. Interviews took place between October 2024 and November 2024, conducted face-to-face in community mental health centers, and lasted an average of 15 minutes. All interviews were audio-recorded with consent, transcribed verbatim, deidentified prior to analysis, and guided by an interview protocol developed based on prior literature and expert consultation. The guide focused on usability, acceptability, user engagement, barriers and facilitators, as well as perceived impacts on medication-related behaviors.

### Participant Recruitment

The study recruited individuals registered in Shanghai’s community mental health system who had been diagnosed with schizophrenia and were receiving maintenance medication. The trial was conducted across 7 community rehabilitation centers administered by the Shanghai Mental Health Center. Shanghai has been recognized as a national leader in the management of schizophrenia, and digital technologies are increasingly being integrated into community-based services to support patient rehabilitation. Eligible participants were formally enrolled after providing written informed consent.

Stable-phase schizophrenia patients were selected as participants by psychiatrists from the Shanghai Mental Health Center. Trained psychiatrists conducted prospective monthly assessments of schizophrenia patients using the Positive and Negative Syndrome Scale (PANSS), with an evaluation period of at least 1 year. Patients were considered to have a stable condition if their total PANSS score did not change by more than 3 points and if there had been no changes in their medication treatment for at least 6 months prior to the neuropsychological assessment [23]. The inclusion and exclusion criteria are summarized in Table 1.

Participants were further required to be in a clinically stable phase, as determined by their PANSS scores.

**Table 1.** Inclusion and exclusion criteria for participant recruitment.

Domain	Inclusion criteria	Exclusion criteria
Registration and diagnosis	Registered in the Shanghai Mental Health Information Management System Diagnosed with schizophrenia according to the *International Classification of Diseases, 10th Revision* and determined to be in a clinically stable phase based on PANSS^a^ assessment	Planning to relocate outside of Shanghai Presence of severe physical illness or organic brain disease Comorbid with other psychotic disorders
Demographics and capacity	Aged 18‐60 y, with at least primary school education Normal or corrected-to-normal vision and hearing Ownership of and ability to independently operate a smartphone or other electronic device	Participation in any intervention or treatment other than medication or basic public health services in the past 6 mo
Treatment status	Receiving maintenance treatment with second-generation antipsychotics.	—^b^
Consent	Participant or family member provided informed consent and signed the consent form.	—

a PANSS: Positive and Negative Syndrome Scale.

b Not available.

### Sample Size Calculation

Sample size was calculated using G*Power 3.1 with a 2-tailed independent samples *t* test. Medication adherence scores were designated as the primary outcome measure. Based on a literature review and pilot study results, the expected between-group mean difference with a pooled SD was 0.78 (0.93), yielding an estimated effect size of approximately 0.83. With a significance level of *α*=.05 and a statistical power of 90%, the required minimum sample size was calculated to be 31 participants per group. Accounting for a 1:1 randomization ratio and a 20% dropout or loss to follow-up rate, the study required at least 76 participants (38 per group). Proportional sampling was conducted across districts based on the number of individuals with schizophrenia in each district.

### Randomization, Allocation Concealment, and Blinding

After providing informed consent, participants were randomly assigned in a 1:1 ratio to the community-based routine rehabilitation group or the digital psychoeducation intervention group using a computer-generated random number table. Randomization ensured that baseline characteristics were evenly distributed between groups. These assessments aimed to evaluate the effectiveness of digital psychoeducation interventions in improving medication adherence and other health outcomes.

### Intervention

#### Application Introduction

This study developed a graphic narrative-based digital psychoeducation application that integrates core concepts from narrative psychology and cognitive-behavioral theory. The intervention aims to enhance medication adherence and treatment motivation among individuals living with chronic-phase schizophrenia. Technologically, the application is grounded in interactive graphics, storyline-driven engagement, and behavioral tracking. It consists of three core modules: narrative storylines, cognitive training games, and self-monitoring logs. The intervention is set within fictional yet realistic life scenarios, allowing for high ecological validity. All user interactions—including choice selections, response times, and behavioral paths—are recorded in real time on the backend for subsequent behavioral analysis and feedback generation. The narrative pathways are dynamically open-ended, meaning that different choices lead to varying outcomes, thereby enhancing immersion and personalization (Multimedia Appendix 1).

#### Narrative Storytelling Psychoeducation

The narrative module is informed by the foundational principle of narrative psychology: individuals construct meaning, integrate experiences, and facilitate behavioral change through storytelling [24]. The application adopts a fictional framework wherein a “nonclinical character supports an individual experiencing schizophrenia,” inviting users to engage in role-playing from a third-person perspective. Users observe the story’s protagonist and assist them in making treatment-related decisions [25]. Although users are not directly narrating their own experiences, they project aspects of their own reality through simulated interactions, enabling externalization of internal struggles and fostering reappraisal of illness and self-identity. This indirect narrative mechanism encourages emotional involvement and character identification, aligning with the dual persuasive processes of *transportation* and *identification* proposed in narrative communication theory, which are known to support behavior change and motivational development [26,27].

To strengthen the educational impact and structural coherence of the storylines, the narratives were designed based on the classic 3-act structure and were iteratively refined under the guidance of psychiatric rehabilitation specialists and clinical psychiatrists. In total, 30 narrative threads were developed, each targeting specific objectives related to medication education (Multimedia Appendix 2).

#### Cognitive Games

The cognitive training game is designed to enhance individuals’ ability to apply cognitive skills in daily life, thereby supporting functional recovery. Codeveloped by clinical psychiatrists and psychiatric rehabilitation experts, this module includes a series of real-world scenario-based tasks—such as grocery shopping or ordering food at a restaurant—that simulate daily challenges and promote skills such as information integration, planning, and decision-making (Multimedia Appendix 2). The games adopt an adaptive learning mechanism in which task difficulty is dynamically adjusted based on the user’s performance. This ensures an optimal balance between challenge and attainability, thereby maintaining engagement and reinforcing learning outcomes.

Beyond strengthening executive function, attention, and problem-solving abilities, this module promotes the transfer of cognitive strategies to real-world environments, offering targeted support for rehabilitation in ecologically relevant contexts.

#### Self-Management Support

The self-management module enables users to schedule medication intake, access educational content related to medication adherence, and input personalized data—such as the daily dosage and timing of medications. The platform facilitates routine tracking of medication usage and provides accessible information on potential side effects and recommended coping strategies. It also includes educational materials highlighting the risks associated with medication nonadherence, including symptom relapse.

Through structured daily logs, users are encouraged to monitor their behavioral patterns, emotional states, and medication routines, thereby fostering self-awareness and self-regulation. This module supports the development of autonomous health behaviors, which are critical for sustaining long-term treatment engagement and improving functional outcomes.

### Outcome Measures

#### Quantitative Outcomes

##### Morisky Medication Adherence Scale

The Morisky Medication Adherence Scale (MMAS) [28-31] comprises eight items. The total score ranges from 0 to 8, with higher scores indicating better medication adherence.

##### Drug Attitude Inventory

The Drug Attitude Inventory (DAI) [32] consists of 10 items, of which 6 are positively scored (correct answers score 1 point and incorrect answers score −1 point), while 4 are reverse-scored (correct answers score −1 point and incorrect answers score 1 point). Higher scores indicate a more favorable attitude toward medication.

##### Social Disability Screening Schedule

The Social Disability Screening Schedule (SDSS) [33] includes 10 items rated on a 3-point scale (0=“no impairment,” 2=“severe impairment,” and 9=“not applicable”). The total score ranges from 0 to 20, with higher scores indicating greater social disability. The evaluation will be performed by trained psychiatric professionals at the time of assessment.

##### Brief Psychiatric Rating Scale

The Brief Psychiatric Rating Scale (BPRS) [34] evaluates 5 factors: anxiety and depression, anergia, thought disturbance, activation, and hostility or suspiciousness. It consists of 18 items scored on a 7-point Likert scale (1=“not present” and 7=“extremely severe”), with a total score ranging from 18 to 126. Higher scores indicate greater symptom severity. The evaluation will be performed by trained psychiatric professionals at the time of assessment.

### Qualitative Outcomes

Participants engaged in semistructured and unstructured interviews accompanied by professional physicians and caregivers. These interviews aimed to explore participants’ attitudes toward the application and its impact on medication adherence. Professional health care providers were also interviewed to evaluate the effectiveness of this kind of intervention. At the end of the intervention, patients were surveyed on their satisfaction with the psychoeducation based on several specific questions (Textbox 1).

Textbox 1. **Semistructured interview guide used to explore usability and user experience of a narrative-based psychoeducational digital intervention.**

Survey on the impact of psychoeducation on medicine adherence (participants)

1. What is the frequency at which the application is utilized?
2. Did you experience any moments during the course of this intervention when you felt the urge to quit up? Why?
3. What motivates or hinders you?
4. Has your medication usage changed as a result of the intervention? How so, if at all. If not, then explain.

Attitudes toward the digital psychoeducation (participants)

1. What is your opinions on such intervention measures?
2. Do you find narratives that are fundamental to be engaging?
3. Which of the chapters that you most impressive?
4. What aspect did you appreciate the most? (scenes, voice-overs, mini-digital psychoeducation interactions, narratives, and characters)

Evaluation of the effectiveness of the digital psychoeducation (mental health professionals)

1. Evaluate the level of patient allure exhibited by the intervention.
2. Kindly assess the efficacy in promoting adherence to medication.
3. In your opinion, which elements are linked to medication adherence? Why?
4. Would you recommend the intervention to your patients?

During each visit, brief semistructured interviews were conducted and audio-recorded to evaluate participants’ attitudes toward the digital psychoeducation. During the baseline visit, interview questions focused on general feedback regarding the digital psychoeducation, including its narrative and visual elements. After the intervention, these questions were revisited, along with additional questions addressing specific digital psychoeducation features, such as the self-management check-in system.

Additionally, backend application usage data were collected, including the date and time of app usage and the story paths chosen within the intervention. Researcher YW analyzed each participant’s experimental sessions by linking the time of app usage to their final story path selections.

### Data Analysis

#### Data Integration

A sequential explanatory mixed methods design was adopted. Quantitative data from the RCT were first analyzed to assess changes in medication adherence, attitudes, and clinical outcomes. Subsequently, qualitative interviews were conducted to explore participants’ experiences and to explain the quantitative results. Integration occurred during the interpretation phase through a triangulation process, in which findings from both data sources were compared and merged to identify areas of convergence, complementarity, and divergence.

#### Quantitative Analysis

All quantitative survey data were analyzed using SPSS version 26.0 (IBM Corporation). Categorical variables were presented as frequencies and percentages, while continuous variables were expressed as means (SD). Comparisons of categorical variables between groups were conducted using *χ*^2^ tests or Fisher exact tests. For continuous variables, independent sample *t* tests or Mann-Whitney *U* tests were used for between-group comparisons, depending on data distribution. Within-group comparisons were performed using paired *t* tests or Wilcoxon signed rank tests. Baseline characteristics between the intervention and control groups were compared using chi-square tests or independent sample *t* tests. The effects of the intervention were evaluated by comparing baseline and postintervention data using independent sample *t* tests or nonparametric alternatives. All statistical tests were 2-tailed, and a *P* value of <.05 was considered statistically significant. No missing data were observed in this study.

#### Qualitative Analysis

The transcripts were analyzed in NVivo (version 12; QSR International) through an inductive form of content analysis [35]. At the beginning of the analysis, DZ and FC extracted text segments from 35 participants’ transcripts to ensure consistency in coding. They then turned to analyze the rest of the transcripts separately. The records were initially coded to gain insights into the participants’ thoughts and behaviors during the intervention process. This involved line-by-line review, extraction of relevant text segments, and identification of codes (n=49), with a focus on participants’ attempts at taking action and their choices. Subsequently, subthemes (n=27) related to different attitudes toward adopting digital psychoeducation to enhance compliance were identified through discussions with participants and experts. The authors then collectively discussed the codes and subthemes, further refining them into broader themes (n=3). Examples of data extraction are listed in Multimedia Appendix 2. This approach allowed us to discern participants’ varying attitudes toward the digital psychoeducation and their implementation of medication practices and processes. Any discrepancies that emerged during the analysis were discussed with the rest of the authors. To ensure the validity of the codes, the authors employed member checking by reaching out to participants to validate interpretations of the data [36]. Specifically, comprehensive results were provided to participants to verify the consistency and accuracy of their experiences with the data.

### Ethical Considerations

This study was conducted in accordance with the ethical principles of the Declaration of Helsinki and the American Psychological Association’s guidelines for research with human participants. The research protocol was reviewed and approved by the Shanghai Jiao Tong University Human Research Ethics Committee (H20230207I). The trial was prospectively registered on ClinicalTrials.gov (NCT06175559). No exemption from ethical review was sought or applied.

All participants, or their legal guardians when appropriate, were provided with detailed verbal and written information about the study procedures, potential risks, and benefits. Written informed consent was obtained prior to enrollment. For participants with limited decision-making capacity, consent was additionally confirmed by a family member or caregiver. The informed consent process covered both primary data collection and the use of deidentified data for secondary analyses without requiring further consent. To protect privacy and confidentiality, all personal identifiers were removed from the study records. The digital psychoeducational application logged only anonymized behavioral data, which were stored in encrypted servers with access restricted to the research team. Interview transcripts were deidentified prior to the analysis. Participants received a gift card valued at approximately US $30 upon completing the study as compensation for their time and effort.

## Results

### Descriptive Analysis

**Figure 2.**
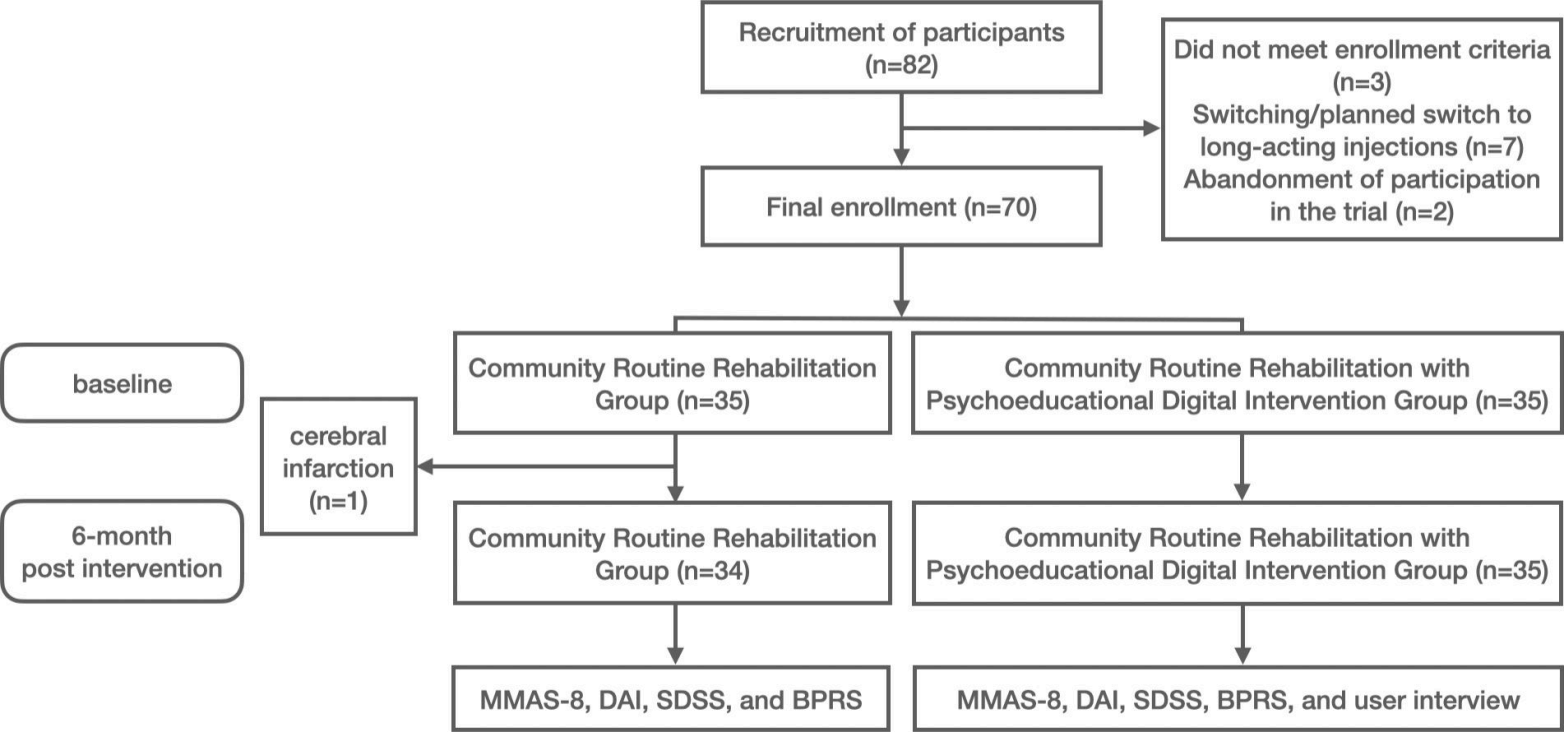
CONSORT (Consolidated Standards for Reporting Trials) flow diagram of participant recruitment of a narrative-based psychoeducational digital intervention. BPRS: Brief Psychiatric Rating Scale; DAI: Drug Attitude Inventory; MMAS-8: 8-item Morisky Medication Adherence Scale; SDSS: Social Disability Screening Schedule.

From March 2024 to October 2024, a total of 82 community-dwelling individuals with schizophrenia undergoing rehabilitation were recruited. Following screening and assessment, 3 participants did not meet the inclusion criteria, and 7 were excluded due to their transition to or planned transition to long-acting injectable medications. The final expected enrollment number was 72 participants (Figure 2). Based on the residential communities of these 72 participants, local intervention sites were identified. Two participants voluntarily withdrew from the study, citing the distance to the intervention site, leaving 70 participants (35 in the community-based rehabilitation group and 35 in the digital psychoeducation intervention group), who completed baseline assessments. During the intervention period, no participants dropped out, and all 70 participants completed the postintervention assessment. The exceptionally low dropout rate can likely be explained by the enforcement of national health policies in China, which require individuals with a confirmed diagnosis of schizophrenia to regularly attend community rehabilitation services as part of their standard care.

### Characteristics of the Study Participants

A total of 70 participants were included in the study, with 43 male patients and 27 female patients. The mean (SD) age of the participants was 44.20 (8.057) years. The distribution of demographic characteristics is shown in Tables2  3. The participants were randomly assigned to either the control group or the intervention group, with 35 individuals in each group. Independent samples *t* test results showed no significant difference in age between the 2 groups (*P*>.05). Chi-square test results indicated no significant differences between the two groups in terms of gender, education level, marital status, or living situation (*P*>.05). Baseline data are provided in Multimedia Appendix 3.

**Table 2.** Baseline demographic and clinical characteristics of participants with stable-phase schizophrenia.

Participants	All (N=70)	Control group (n=35)	Experimental group (n=35)	χ^2^/*t* test^a^ (df)	*P* value
Sex, n				0.54 (1)	.46
Male	43	20	23		
Female	27	15	12		
Age (y), mean (SD)	44.20 (8.06)	44.29 (9.10)	11 (6.99)	0.09 (68)	.93
Education, n				3.29 (2)	.19
Primary school	2	0	2		
Middle school	45	21	24		
College or above	23	14	9		
Marital status, n				0.51 (2)	.77
Married	11	5	6		
Divorced and widowed	10	6	4		
Unmarried	49	24	25		
Residence status, n				0.22 (1)	.64
Living alone	5	3	2		
Living with family	65	32	33		

a *t* tests were used for continuous variables (eg, age), and chi-square tests were used for categorical variables (eg, gender, education level, marital status, and residence status).

**Table 3.** Demographic characteristics of two participating mental health professionals.

Mental health professionals	Major	Gender	Age (y)	Years of experience
D1	Nursing	Female	39	15
D2	Public health management	Female	32	6
D3	Public health management	Male	41	12
D4	Psychology	Male	30	2
D5	Psychology	Female	42	15

### Analysis of Intervention Results

This study evaluated differences between the intervention and control groups across multiple outcome measures, including medication adherence, attitudes toward medication, social functioning, and clinical symptoms (Table 4). The intervention group demonstrated significant improvement in medication adherence. At postintervention, a statistically significant between-group difference was observed, with a mean difference of 1.27 (95% CI 0.30-2.24; *P*=.02). Regarding attitudes toward medication, the intervention group also showed a significant improvement compared to the control group, with a mean difference of 3.41 (95% CI 1.18-5.65; *P*=.002).

**Table 4.** Changes in medication adherence, medication attitude, social functioning, and clinical symptoms from baseline to 6 months in a randomized controlled trial.

Characteristics	Intervention group, mean (SD)	Within-group *P* value	Control group, mean (SD)	Within-group *P* value	Mean difference (95% CI)	Between-group *P* value
Medication adherence		.03^a^		.11	1.27 (0.30 to 2.24)	.02^a^
Pre	6.91 (1.66)		7.23 (1.22)			
Post	7.66 (0.49)		6.68 (2.05)			
Medication attitude		<.001^b^		.47	3.41 (1.18 to 5.65)	.002^a^
Pre	4.97 (3.64)		5.83 (3.12)			
Post	8.91 (1.90)		6.35 (3.89)			
Social functioning		.03^a^		.85	−1.91 (−8.55 to 4.73)	.34
Pre	2.20 (2.83)		2.20 (2.79)			
Post	1.06 (1.97)		2.88 (3.68)			
Clinical symptoms		.06		.66	−7.88 (−34.90 to 19.14)	.12
Pre	28.37 (16.04)		25.77 (11.08)			
Post	21.37 (3.49)		26.68 (8.48)			

a *P*<.05.

b *P*<.001.

### Qualitative Feedback and User Experience

Among the secondary outcomes, participants in the intervention group exhibited a notable within-group improvement in social functioning (*P*=.03). However, no statistically significant between-group differences were found in social functioning or clinical symptom severity (*P*>.05).

#### Application Usage (Back-End Data)

Analysis of back-end data indicated that 34 of 35 (97.14%) participants completed all game content. However, participants exhibited relatively high error rates in decisions related to certain topics, highlighting areas for targeted education in future interventions.

#### Alcohol Consumption and Medication

Although 25 of 35 (71.43%) participants selected the correct response—“Alcohol should be avoided, as it interacts with antipsychotic medications causing central nervous system depression, and excessive drinking may trigger other severe drug side effects”—a notable proportion (13/35, 37.14%) chose incorrect options, such as “All medicines are toxic, better not take them at all” or “Life is short, it’s fine to skip once in a while.” This suggests persistent misconceptions regarding alcohol–medication interactions.

#### Medication When Going Out

Some participants (8/35, 22.86%) initially chose unsafe options when deciding whether to take medication while going out, including “Skipping occasionally is fine” or “It’s okay to skip while traveling.” After experiencing negative story consequences, participants corrected their choices to the appropriate response: “Ensure to bring and take medications on time to avoid missed doses.”

#### Medication Side Effects

Most participants (31/35, 88.57%) correctly responded to in-story side effects by selecting “Consult a professional promptly if experiencing discomfort.” Nevertheless, a subset (7/35, 20%) initially chose the incorrect response, “It’s just lack of exercise, so I can endure it.”

Overall, these back-end data reveal both high engagement with the application and specific knowledge gaps, particularly regarding alcohol use, medication adherence while traveling, and interpretation of side effects. These findings underscore key areas for future educational emphasis.

### Result of the Interview

#### Overview

The quantitative analysis revealed that the intervention significantly improved medication adherence and attitudes toward medication compared with standard community rehabilitation. The qualitative findings complemented these results by illustrating how narrative immersion and interactive gameplay enhanced users’ motivation and engagement, thereby reinforcing the behavioral changes observed in the quantitative phase. The demographic and clinical characteristics of the respondents are shown in Table 2. Thematic saturation was achieved after approximately 25 interviews, when no substantially new codes emerged. The remaining 10 interviews confirmed and enriched existing themes, particularly regarding variations in family support and illness duration. The specifics of the qualitative findings are displayed in Multimedia Appendix 4. Three main higher-order themes emerged from the participants’ and experts’ accounts (Multimedia Appendix 5).

#### Adherence and Usability

This theme encompassed factors related to medication adherence and the usability of the narrative-based digital intervention. Adherence was strengthened through increased medication knowledge, enhanced confidence in recovery outcomes, and active engagement with interactive narratives. Participants generally reported that the narrative intervention promoted adherence by enhancing knowledge about medication and building awareness of illness consequences, going beyond the functions of traditional reminder tools. This cognitive restructuring process appeared to activate intrinsic motivation. One participant explicitly noted that the intervention content changed their pattern of avoiding medication: “I used to avoid taking my medication due to side effects. After using this app, I realized that failing to reach the prescribed dose could have real consequences. Now I remind myself more consistently to follow the plan.” Another participant stated, “I used to reduce doses or skip medication on my own… but now I feel more capable of managing my condition and trusting my treatment plan.”

Positive reinforcement, such as the perceived “increase in medication knowledge” and “improvement in health status” after completing each narrative scenario, significantly enhanced participants’ self-efficacy and encouraged continued engagement. Additionally, the intervention’s self-management interface, which clearly displayed daily progress in medication-taking and task completion, provided ongoing external motivation: “Every time I log in, I feel like I’m working toward my health, which really motivates me.” However, feedback tone and clarity were sensitive factors that could influence continued engagement. When participants made incorrect choices, the perceived harshness of outcomes sometimes triggered overstimulation and negative emotions, leading to tension and feelings of being scolded (“A bad ending made me angry, and I didn’t understand why I was wrong”), highlighting the importance of strategic and tactful feedback.

#### Experiences and Attitudes Toward the Digital Psychoeducation

This theme revealed that participants expressed positive attitudes toward the narrative-based intervention, particularly appreciating its novelty and immersive quality. They described this learning approach as “not like a class, more like living inside a story,” which significantly enhanced the learning experience. High acceptance was closely related to cultural sensitivity in the intervention, such as familiar home environments, clothing styles, and locally grounded story backgrounds. Participants noted, “The character design feels like people I really know… it is very relatable and comforting.” Psychologically, the narrative also provided participants with important emotional connection and a sense of inclusion. Some participants reported feeling socially isolated in real life, but nonplayer characters within the intervention offered companionship and acceptance: “I always feel like people around me don’t like me or want to talk to me… only they (in the app) are willing to talk to me.”

Despite the overall cultural relevance of the narrative content, limitations remained at the individualized level. Some participants indicated that the narrative did not fully reflect their specific clinical or demographic characteristics, resulting in reduced relevance for certain content. One male participant noted, “Not all themes relate to my personal experiences, and not all side effects happened to me,” suggesting that highly themed or specific content may create cognitive distance or misalignment with other user groups.

#### Expectations for the Digital Psychoeducation

This theme explored participants’ suggestions regarding design elements and interactive features of the intervention, aimed at enhancing understanding of medication use. While initial experiences were positive, both participants and therapists emphasized the importance of ongoing content updates and functional completeness for maintaining long-term adherence. One participant remarked, “After completing all the stories, there’s nothing new. I hope it could include more everyday situations, like working or traveling with friends.” Due to the relatively simple content, one participant completed all narrative scenarios within 6 weeks, resulting in diminished interest later. The rapid consumption of content led some participants to discontinue usage after story updates ceased: “Towards the end, only the daily check-in feature was available, no new storylines. So, I just stopped engaging with the intervention.”

From a functional perspective, participants repeatedly emphasized the need for external reminders, not only for new story releases but also for synchronized medication taking, requesting that the app “send notifications like other apps.” Therapists highlighted the feasibility of future technological integration, suggesting “It would be great if AI could generate more interactive stories rather than having doctors spend time creating scripts,” to ensure a continuous content supply and reduce the burden on clinical staff, thereby enabling sustainable integration of the system. Finally, some participants noted frustration with the lack of a review function: “Sometimes I have to interrupt the narrative due to other things happening, and I forget what happened. When I come back to the story, I realize that progress from before that level wasn’t saved…” indicating the need to optimize memory and review mechanisms.

## Discussion

### Principal Findings

This study evaluated the usability, user experience, and behavioral effects of a narrative-based digital psychoeducational intervention developed for individuals with chronic schizophrenia in community settings. Using a mixed methods design, our findings suggest that, compared with standard community rehabilitation, the intervention significantly improved medication adherence and attitudes toward medication, while no significant changes were observed in clinical symptoms. Complementary qualitative findings further highlighted that participants valued the narrative elements for enhancing knowledge, motivation, and engagement, while also reporting challenges such as negative emotional reactions to corrective feedback. Most participants emphasized that maintaining long-term content engagement remained a concern, underscoring the need for future digital designs to incorporate personalized, accessible, and supportive features.

Specifically, following the narrative-driven digital psychoeducational intervention, participants exhibited notable improvements in medication adherence and medication attitude. Qualitative interviews provided contextual explanations for these quantitative findings, illustrating how the intervention influenced behavior through narrative mechanisms. Immersive storylines and interactive feedback enhanced participants’ understanding of medication routines and their sense of control, leading to better adherence and fewer unplanned treatment interruptions. For example, some users mentioned that after completing the story, they “felt responsible for the character’s health,” reflecting a motivational shift from passive medication-taking to active self-management. This indicates that the digital format not only increased engagement but also fostered emotional resonance and cognitive involvement, supporting the observed behavioral improvements [37,38]. Care providers noted that integrating the intervention into repetitive cognitive rehabilitation sessions encouraged sustained participation in regular health-promoting behaviors. These improvements may be attributed to embedded mechanisms of emotional engagement and cognitive participation within the system [39]. Consistent with prior research, digital technologies such as smartphone applications have been found acceptable and feasible for individuals with psychotic disorders [40]. Existing mobile applications targeting medication adherence in schizophrenia mainly address logistical and memory-related barriers through reminders, dose tracking, and external reinforcement [41,42]. However, for individuals with schizophrenia, key barriers to treatment adherence are often internal, psychological, and cognitive, such as limited insight, misconceptions about side effects, perceived stigma, and reduced intrinsic motivation for long-term therapy [43]. Thus, interventions targeting patients’ deeper cognitive representations of illness and treatment offer a promising direction for narrative-based approaches [44]. Immersive narratives provided a judgment-free, low-pressure environment in which participants could temporarily shift from the passive patient role to that of an active, rational decision-maker, engaging in simulated choices about medication and recovery. Many participants perceived the scenarios as familiar and personally relevant, which appeared to reduce resistance toward illness-related content and strengthen their willingness to engage in medication behaviors [41,42]. Although no significant between-group differences were observed in clinical symptoms, within-group improvements were identified. Qualitative narratives illuminated these patterns, as some participants described transient negative emotions during feedback or difficulties sustaining motivation over time, suggesting that behavioral and attitudinal improvements may precede measurable symptom changes [43,44]. Also, given the long-term trajectory of schizophrenia recovery, these findings collectively suggest that increased persistence and motivation could represent intermediate mechanistic changes that may facilitate long-term clinical benefits [45-47].

Regarding user experience and attitudes, most participants expressed positive feedback toward the modular narrative structure, describing the intervention as immersive and engaging. These qualitative findings complemented the quantitative results on adherence and medication attitude, suggesting that enhanced engagement and motivation may have mediated the observed behavioral improvements. By framing participants’ self-representation as part of a healthy population, the interactive design reduced perceived stigma and promoted active knowledge acquisition and health behavior. Therapists noted that the intervention embedded medication-related knowledge through a sequence of stories, situational decision points, and real-time feedback, enabling participants to rehearse treatment-related decisions in a safe, low-pressure environment [48]. This narrative-based approach not only facilitated knowledge acquisition but also preserved users’ autonomy and sense of realism. The usability and emotional engagement of the intervention appear to underpin the cognitive and motivational mechanisms that supported adherence improvement. As Stephens [49] emphasized, narratives serve not only as a means of organizing experience but also as a tool for identity construction and social positioning. By persuading a virtual character to “take medication on time,” participants effectively constructed a socially valued identity—one characterized by cognitive competence and responsibility. This process represents a form of narrative repositioning, in which individuals psychologically shift from passive recipients of care to active agents of self-management, thereby enhancing perceived control and treatment motivation [50].

However, some users reported that certain narratives reflecting specific cultural or gendered contexts limited identification and engagement, highlighting the need for more personalized and culturally adaptive content in future iterations. Additionally, motivational activation was influenced not only by the narrative content but also by functional design elements such as pacing, reward systems, and feedback mechanisms. Participants reported confusion when storylines became overly abstract or fragmented, particularly during periods of cognitive fatigue, emphasizing the importance of clarity and simplicity in interface design for individuals with psychotic disorders. Thus, in addition to ensuring psychological relevance and cultural appropriateness, system design must prioritize simplicity and error tolerance [48]. Future development should continue to involve diverse stakeholders—including therapists, patients, and family members—for iterative discussion and codesign [51]. Mechanistically, participants indicated that the cognitive game components were seamlessly integrated with the narrative elements, enabling repeated practice in recognizing and reframing unhelpful automatic thoughts [42]. The task-based structure and immediate feedback combined behavioral activation with cognitive restructuring, helping participants modify maladaptive thinking patterns and rebuild medication-related cognitive frameworks. This mechanism aligns with the principles of cognitive behavioral therapy, which seeks to enhance adherence by reducing cognitive resistance and emotional conflict while strengthening behavioral consistency and motivation [52,53]. Finally, achievement-based feedback, milestone rewards, and task-driven structures created a sustained motivational environment that supported engagement even when immediate reinforcement was limited [54].

This mixed methods study provides preliminary evidence that the narrative-based digital psychoeducational intervention tested in this study demonstrated good usability and acceptability and may support short-term improvements in medication adherence and attitudes toward medication among individuals with chronic schizophrenia. Although short-term symptom outcomes did not show significant improvement, the intervention addressed key behavioral and motivational processes critical for long-term rehabilitation. These findings emphasize the potential of this narrative-based, culturally contextualized digital tool to complement traditional community rehabilitation and to inform the development of more sustainable models of psychiatric care. Future research should extend follow-up periods to examine delayed clinical effects, incorporate adaptive and AI-driven narrative content to enhance cultural applicability, and test scalability across different health systems and populations.

### Limitations

This study has several limitations. First, the financial incentives provided to participants may have facilitated their continued engagement. However, the primary purpose of these incentives was to compensate participants for their time and travel expenses. The amount of compensation was limited and unlikely to produce long-term changes in medication-taking behavior, suggesting that observed improvements in adherence are more likely attributable to the intervention mechanisms themselves. Nevertheless, we acknowledge that financial incentives may have modestly increased engagement, representing a potential confounding factor. Future studies could consider designing conditions without incentives or employing stratified analyses to control for this potential confounder. Second, all outcome measures relied on self-report, introducing the risk of subjective bias. The involvement of experienced psychiatrists may help mitigate such bias. Third, participants’ familiarity and comfort with using smartphones to play the game varied, which could have influenced the effectiveness of the intervention. Additionally, as individuals in the intervention group were aware that they were receiving a digital intervention aimed at improving medication adherence, the absence of participant blinding may have introduced expectancy bias. Finally, while cultural customization likely enhanced the acceptability of the intervention within Chinese community settings, it may limit generalizability to more diverse or international populations. Future iterations of narrative-based psychoeducation should consider incorporating cross-cultural adaptation processes or leveraging AI-driven adaptive narratives to dynamically generate culturally relevant storylines.

Despite these limitations, the findings offer meaningful insights into the potential of narrative-driven digital psychoeducation to enhance medication adherence among individuals with schizophrenia in the long-term chronic phase. The intervention’s integration of immersive storytelling, cognitive training, and self-management features presents a promising direction for future psychosocial support tools.

### Conclusion

Our mixed methods findings indicate that the narrative-based digital psychoeducational intervention developed in this study demonstrated good usability and acceptability among individuals with chronic schizophrenia and may support short-term improvements in medication adherence and attitudes toward medication. Mobile health interventions of this kind could serve as a valuable complement to traditional community rehabilitation by enhancing behavioral and motivational mechanisms that contribute to long-term recovery. Nevertheless, careful attention should be paid to feedback design and cultural adaptation of narrative content, and tasks should be tailored to users’ preferences and cognitive characteristics to ensure sustained usability and long-term effectiveness of the intervention.

## Supplementary material

10.2196/59175 Multimedia Appendix 1 Video of Healing Town.

10.2196/59175 Multimedia Appendix 2 Content introduction of Healing Town.

10.2196/59175 Multimedia Appendix 3 Data at baseline.

10.2196/59175 Multimedia Appendix 4 Themes of the interview.

10.2196/59175 Multimedia Appendix 5 High-order themes emerged from narrations.

10.2196/59175 Checklist 1 CONSORT-EHEALTH checklist (V 1.6.1).

## Abbreviations

BPRS: Brief Psychiatric Rating Scale
CONSORT: Consolidated Standards of Reporting Trials
CONSORT-EHEALTH: Consolidated Standards of Reporting Trials of Electronic and Mobile Health Applications and Online Telehealth
DAI: Drug Attitude Inventory
MMAS: Morisky Medication Adherence Scale
PANSS: Positive and Negative Syndrome Scale
RCT: randomized controlled trial
SDSS: Social Disability Screening Schedule
