# Supplementary material for: Assessing Usage and Usability of a Narrative-Based Psychoeducational Digital Intervention to Improve Medication Adherence Among Individuals With Schizophrenia in a Stable Phase: Mixed Methods Study
Source: J Med Internet Res. 2026 Jan 20;28:e59175. doi: 10.2196/59175 (PMC12818729; doi:10.2196/59175)
Supplement: Multimedia Appendix 2 [file jmir-v28-e59175-s002.docx]

Appendix file 1

The prototypes developed in this study were primarily built using Unity version 2021.3.26f1c1, with the visual scripting tool Bolt used for front-end interface and interaction design. Bolt was chosen to streamline the development process and enable more efficient implementation of user interactions and interface features. In parallel, a basic data storage system was integrated to ensure the prototype's stability and effective data management. All output data generated by the prototype is saved in Excel format. Specifically, the application "Healing Town" records each user's selection time and the items they choose upon entry. This data is stored in Excel to facilitate easy download and further analysis by the research team.

**1.Digital Psychoeducation Intervention Features**

The project development team created an application "Healing Town," with each unit lasting 20 minutes (including narrative and cognitive games), totaling 30 stories. These narrative scenarios are embedded in an intuitive user interface accessible via Android-based devices (Figure 1). As the story progresses, characters express emotions like joy, anger, and sadness. The team selected suitable voice tones for each character, and TTS voiceovers were created. Careful attention was given to ensure the characters' actions, behaviors, and movements provide patients with a realistic experience.


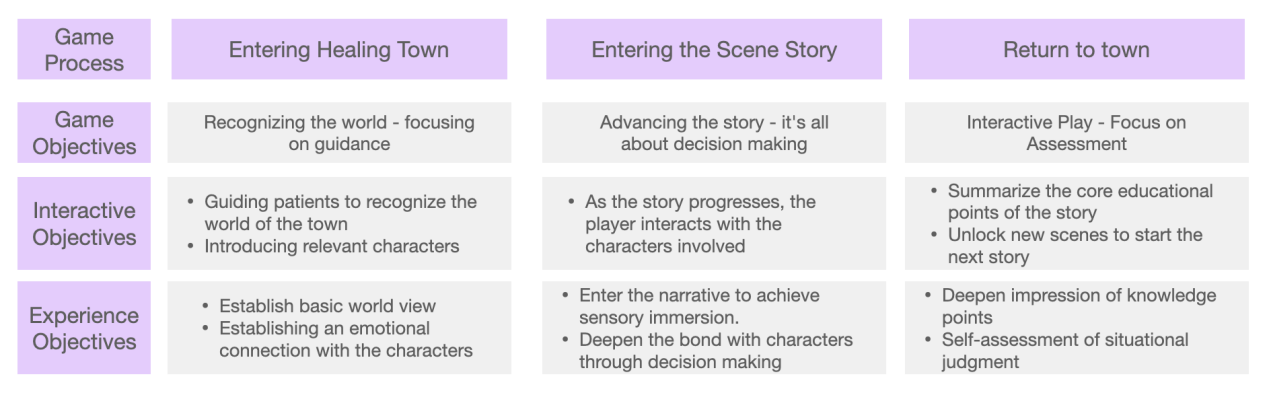


Figure 1: Usage of Healing Town

The first part is the narrative story (Figure 2). The narrative game is highly immersive, creating a virtual world where patients (players) play the role of the character "JIANGUO" make everyday decisions. For example, when a character experiences medication side effects or contemplates eating unhealthy food, the player must choose how to respond. Figure 1 illustrates how the player, acting as a friend (JIANGUO), advises "Lao Wang" to avoid unhealthy food. The aim is to promote healthier behavior in both the character and the player.


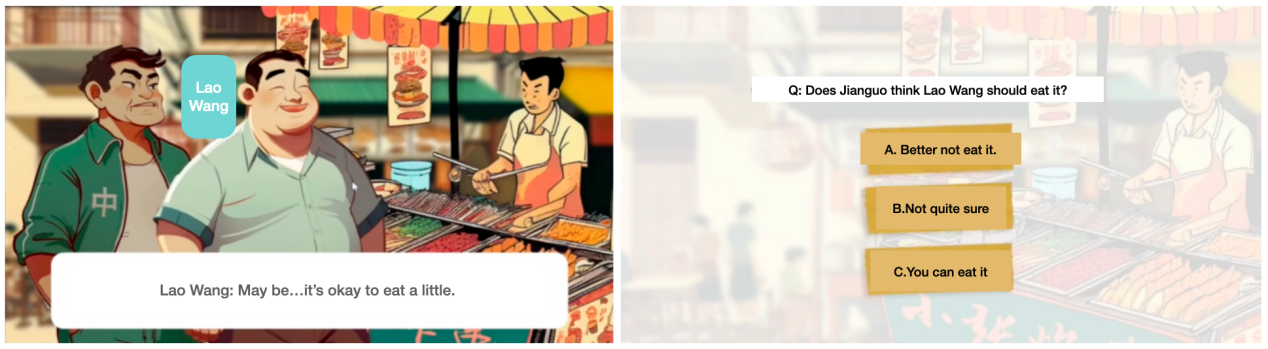


**Figure 2**: Screenshots of the narrative game.

**Figure 3** depicts a scenario where Lao Wang's friends invite him to drink alcohol. Players need to help Lao Wang politely decline. These stories aim to restore patients' social functions, reinforcing healthy decision-making through their interaction with characters. Scripts for the 30 scenarios were written by doctors at the Shanghai Mental Health Center, with different storylines based on patients' choices. The narrative ties directly into cognitive tasks, merging cognitive training with real-life scenarios.


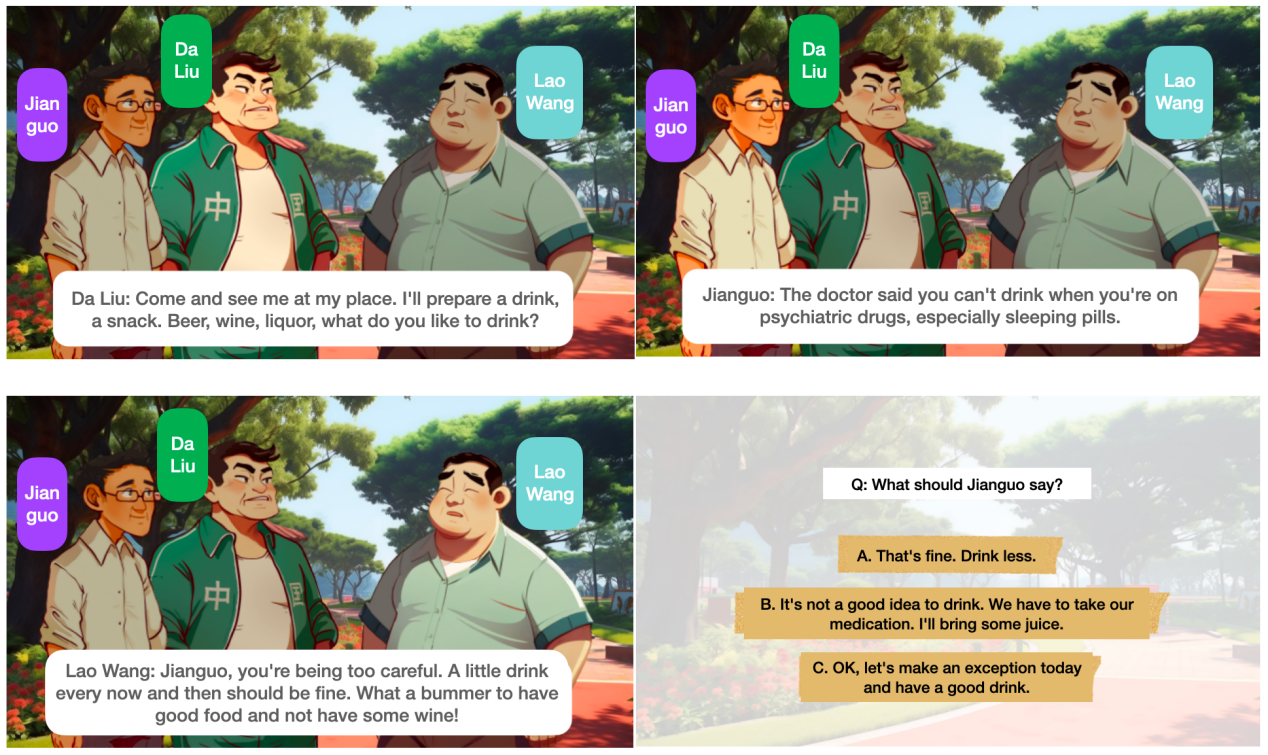


**Figure 3**: Screenshots of the narrative game.

**Cognitive Games** (Figure 4) In this game section, players face quick, interactive mini-games that involve choosing appropriate actions, like managing diet (avoiding spicy, greasy, or sweet foods) and selecting daily exercises (avoiding intense activities).


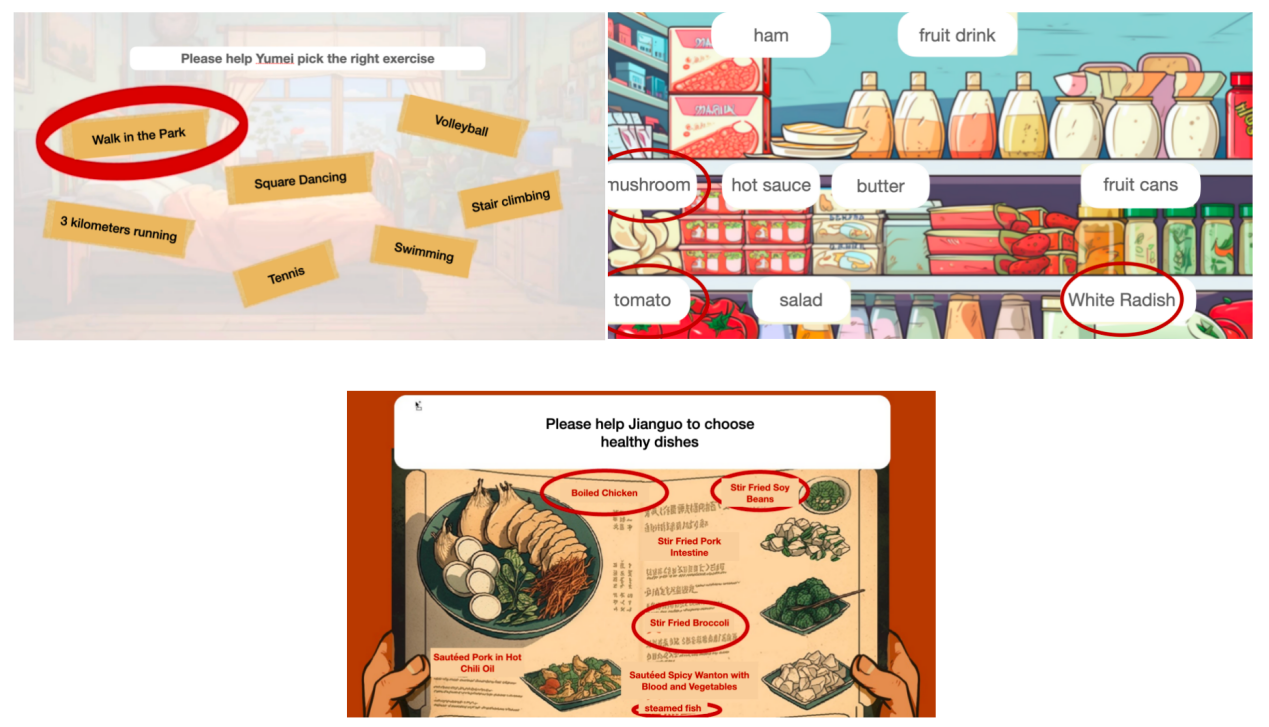

**Figure 4**: Screenshots of cognitive training games.

**Self-Management** (Figure 5) The application allows users to input medication data prescribed by doctors, such as dosage and administration intervals, with reminders for when to take their medication. It also offers comprehensive information on medication side effects and coping strategies. Users are prompted to record their medication intake and provided with information on the negative consequences of non-adherence, such as relapses.


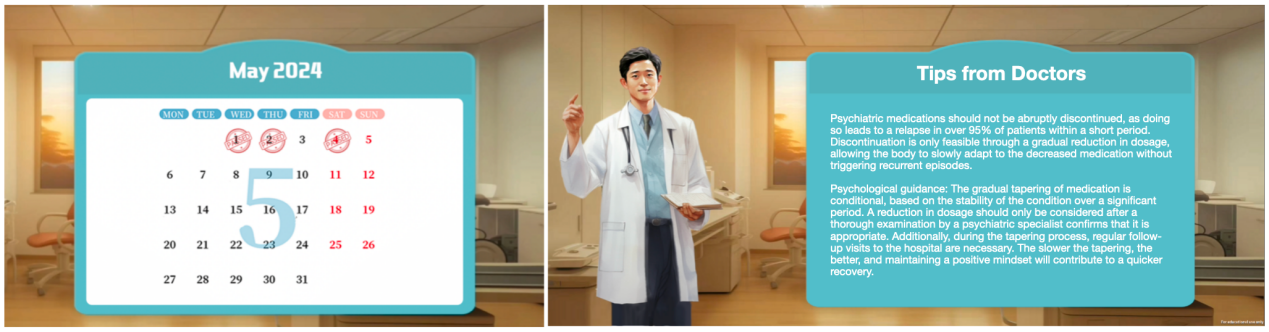

**Figure 5**: Screenshot of self-management tips and tracking features.

**2.Interaction with the application**
Before the intervention, therapists and researchers explain the interaction methods to both patients and their caregivers (Figure 6). Throughout the intervention, participants are required to attend at least three weekly in-person sessions supervised by a therapist, each session lasting approximately 20-30 minutes. During this time, they complete one unit of the story, discussing the relevance of the story and its connection to real life with the therapist. This helps patients better remember the medication knowledge.


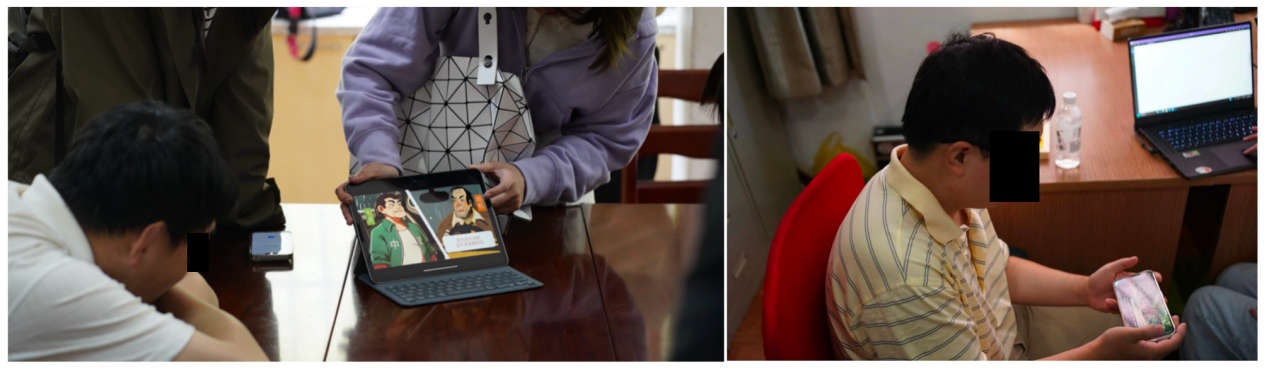

**Figure 6**: Pre-intervention therapist session.
