# Supplementary material for: Assessing Usage and Usability of a Narrative-Based Psychoeducational Digital Intervention to Improve Medication Adherence Among Individuals With Schizophrenia in a Stable Phase: Mixed Methods Study
Source: J Med Internet Res. 2026 Jan 20;28:e59175. doi: 10.2196/59175 (PMC12818729; doi:10.2196/59175)
Supplement: Multimedia Appendix 3 [file jmir-v28-e59175-s003.docx]

Appendix file 2 Characteristics of study participants in the control and intervention groups at baseline

| Variable | Control group (N=35) | Intervention group (N=35) | t | p |
| --- | --- | --- | --- | --- |
| Medication Adherence | 7.229±1.222 | 6.907±1.657 | 0.923 | 0.359 |
| Attitude toward taking medication | 5.83±3.120 | 4.97±3.642 | 1.057 | 0.294 |
| Social functioning | 2.20±2.795 | 2.20±2.826 | <0.001 | 0.999 |
| Clinical symptoms | 25.77±11.083 | 28.37±16.042 | -0.789 | 0.433 |
