# Supplementary material for: Assessing Usage and Usability of a Narrative-Based Psychoeducational Digital Intervention to Improve Medication Adherence Among Individuals With Schizophrenia in a Stable Phase: Mixed Methods Study
Source: J Med Internet Res. 2026 Jan 20;28:e59175. doi: 10.2196/59175 (PMC12818729; doi:10.2196/59175)
Supplement: Multimedia Appendix 4 [file jmir-v28-e59175-s004.docx]

| Themes | sub-themes | codes | Condense meaningful segments | Meaningful text fragments |
| --- | --- | --- | --- | --- |
| Adherence and usability | Personal and Environmental Factors | Personal Factors | Influence medication adherence: beliefs, attitudes, knowledge.  Some reduce/discontinue medication due to distress or misunderstanding.  Digital psychoeducates on medication adherence, boosting confidence in recovery.  Anticipation of storyline drives engagement, altering behavior. | I used to feel uncomfortable after taking medication, so I didn't really want to take it. But this game showed me that not taking my meds is actually bad for my condition.  It made me see the consequences of stopping my medication and taught me how to take it correctly.  I used to reduce the dose or stop taking it on my own……but now I feel like I can manage my condition better and trust my treatment plan more. |
|  |  | Environmental Factors | Stories' outcomes provide immediate feedback for real-world decisions.  Simulates social contexts of psychosis, aiding behavioral understanding.  Environments mimic real-life, aiding in adapting to challenges.  Player interactions and role models inspire development and effort. | In the game, there are characters who go through the same condition I do, and their choices affect me.  The doctors in the game will explain the benefits of taking my medication and the consequences of stopping it.  I'm really looking forward to the next updates for the game... |
|  | Digital Psychoeducation Setting | Realistic Interaction Scenarios | Majority found stort content easy to understand with minimal introduction.  Cognitive game segment required concentrated attention but was manageable with appropriate challenge.  Showing the conflict between difficulty and actual useage | I hope that my friends could benefit from the game. I and my friends can understand easily how to play with each other in the game...  When their condition gets worse, I feel really sad too...I empathize with their situation..  The part “choose groceries” is something I can actually use in my daily life...  The game is interesting……but I didn't follow the rule of completing one day's content each day. Instead, I completed several sessions one day.  Since the game is relatively easy, I finished most of the content pretty quickly. |
|  |  | Emphasis on forms of feedback | Limited impact of feedback on adherence.  Caution needed in tone and manner of prompts for incorrect choices.  Explicit or severe prompts may cause emotional distress, while tactful feedback aids comprehension and learning.  Self-administration features help remind participants of medication-taking behaviors | A bad ending made me angry, and I didn’t understand why I was wrong.  Even when I made wrong choices during the game, there was no feedback…….  The interface clearly showed me my daily tasks, including taking my medication and completing certain in-game challenges. Every time I logged in, I felt like I was making an effort for my health, which really motivated me.  I found the self-management interface in the game very helpful. It reminded me to log in every day, so I wouldn't forget to take my medication." |
| Experiences and Attitudes Toward the application | Attitude Towards the Approach | Initial Reception | Participants found the approach novel and were eager to try it.  Content derived from daily life enhances relevance and appeal. | I think this approach is really innovative. I never thought treatment could be so fun……really looking forward to the final version.  The previous treatment methods were often boring and took a lot of time or effort... |
|  |  | Cultural Sensitivity | Recognition of scene style and content design enhances acceptability.  Greater cultural sensitivity improves relevance and appeal to intended audience. | The stories incorporate a lot of realistic elements, like unique sports clothing and the home decor from the last century, which brings back a lot of memories.  The character designs feel like people I really know……it very relatable and comforting. |
|  |  | Negative Emotions | Cues for incorrect decisions may lead to negative emotions.  Anxiety, frustration, or confusion may discourage continued use.  Negative feedback or fear of errors may hinder participation and impact overall experience. | I know the game is supposed to help me, but when I make a wrong choice, I still get a bit nervous...  I don't like being pointed out for my mistakes... If I make another mistake when I try again, I just don't feel like continuing. |
|  | Attitude Towards the Components | Mapping of story scenarios to real life | Ensure that story content reflects a wide range of cultural backgrounds and values.  Design with patients of different physical abilities and cognitive abilities in mind.  Consider the differences between urban and rural environments and ensure that scenarios and plots reflect multiple geographic contexts. | The “ikebana story” has a really good ending. I hope that when I get better, I can join in activities with my friends again... The knowledge in this story is really helpful for me, it reduces my anxiety.  Many scenes and situations in the game are things I encounter in my daily life, which makes me feel familiar and makes it easier for me to get involved. |
|  |  | Mapping of game scenarios in the self-healing process | A healthcare professional observes the content of stories written by patients in their daily lives。  Combined with the visual expression of the story makers, presenting a work that resonates with patients. | I like the theme of the story about women experiencing hormonal changes due to anti-psychotic medication because I actually have this hormonal issue... This story makes me feel understood.  Through these stories, I learned that patients undergoing anti-psychotic treatment may experience physical changes... It makes me feel less anxious about it. |
|  | engagement | Immersion | Promote players' emotional attachment to game characters | In real life, I feel isolated. But in the game, it's different... The characters in the game are just like real people.  I used to have a friend who looked like Lao Wang... but we lost touch after I got sick.  I feel like I'm communicating with them..." |
|  |  | Inclusion | The game's characterization succeeds in creating friends for whom there is no discrimination | I always feel like the people around me don't like me and don't want to talk to me... Only them (the people in the game) are willing to talk to me.  I don't like going out or talking to others... They think there's something wrong with my mind. The NPC here...they are my friends. |
| Expectations for the application | Digital Psychoeducation Content | Continuous Updating | Content must evolve to match participant expectations.  Initial interest wanes over time, necessitating more intricate narratives to sustain engagement. | After finishing all the story content now, there are no more updates...  Is there anything story that is about taking a taxi, having dinner with friends, or working out? |
|  |  | Impact on Medication Use | Some participants find the recordings mildly motivating for medication adherence. | Towards the end, only the daily check-in feature was available, no new storylines. So, I just stopped playing the game.  Could you add more to the mini-games section (like cognitive games)? The ones now are getting quite boring. They gave me 8 weeks for this, but I finished it all in 4 weeks. |
|  | Digital Psychoeducation Function | The need for reminders | Users would like to receive reminders about tasks, activities, or important updates, etc. | I hope you can remind me promptly when there are new stories... Also, for the daily medication, I need a reminder. Actually, I do take my medicines, I just forget to synchronize that in the game sometimes.  Can you send a notification like what other apps do? |
|  |  | Mechanisms for motivation enhancement | Lack of motivation to adhere to self-management | It seems like there's not much loss if I don't log in...  When I see my log-in record, it gives me the motivation to keep going... Maybe I'm a bit “OCD”, I only feel satisfied when I see the log-in page filled up.  I also hope the game can add more interactive features, like online quizzes, group discussions, and such. |
|  |  | Need for review | Review function to improve application consistency | Sometimes I have to interrupt the narrative story due to other things happening, and I forget what happened, then when I come back to the story, I realize that the progress before that level wasn't saved...  I often forget the storyline I've played, which can be frustrating. |
