# Supplementary material for: Assessing Usage and Usability of a Narrative-Based Psychoeducational Digital Intervention to Improve Medication Adherence Among Individuals With Schizophrenia in a Stable Phase: Mixed Methods Study
Source: J Med Internet Res. 2026 Jan 20;28:e59175. doi: 10.2196/59175 (PMC12818729; doi:10.2196/59175)
Supplement: Multimedia Appendix 5 [file jmir-v28-e59175-s005.docx]

Appendix file 4 High-order themes emerged from narrations with 35 schizophrenia patients and 5 mental health professionals

| **Adherence and usability** |
| --- |
| Personal and Environmental Factors Interviews with both patients and clinicians revealed that a combination of personal and environmental factors influenced the effectiveness of the digital psychoeducation intervention on medication adherence. Among these, personal factors were particularly prominent, including beliefs, expectations, attitudes, and knowledge about the illness and pharmacological treatment. Some participants reported modifying or discontinuing their medication regimen due to a lack of understanding of their condition or subjective perceptions of physical discomfort. The digital psychoeducation employed an implicit narrative approach to deconstruct the risks of such non-adherent behaviors, while simultaneously emphasizing the benefits of following prescribed treatment plans. This helped enhance users’ understanding and acceptance of health-promoting behaviors. When participants were exposed to scientifically grounded recovery knowledge within the narrative context, their confidence in treatment outcomes increased, leading to more positive attitudes. In some cases, anticipation about the unfolding storyline further enhanced emotional engagement, which, in turn, deepened their involvement in the cognitive training process and subtly contributed to improved medication-taking behaviors. |
| Beyond individual factors, a smaller number of participants highlighted the influence of social and physical environmental factors. The intervention’s real-time feedback mechanisms helped users understand the consequences of their actions, enabling more informed decision-making in daily life. Simulated social environments—such as family or workplace scenarios—were described as helpful in preparing users to address real-world challenges. In addition, observing the behaviors of others and interacting with peers within the intervention offered participants new behavioral strategies and emotional regulation techniques. The presence of positive role models within the system served as motivational anchors, reinforcing behavioral change and improving adherence. These observations reflect mechanisms consistent with social learning theory, where learning is facilitated through behavioral feedback and observational modeling. |
| **Digital Psychoeducation Setting** Participants generally perceived the content of the digital psychoeducation as accessible and easy to understand. The daily narrative tasks were embedded in realistic, everyday contexts that allowed users to make decisions and judgments without significant demands on linguistic or logical reasoning. By contrast, the cognitive training modules placed higher demands on attention and information-processing abilities. Most participants reported that these modules were appropriately challenging and contributed to sustained engagement. Regarding the feedback mechanisms, some participants noted that the tone and phrasing of prompts significantly affected their user experience. Specifically, feedback that was overly direct or critical could evoke negative emotional responses and reduce willingness to participate. In contrast, prompts that were phrased in a gentle and supportive manner were seen as more effective in maintaining a positive learning mindset and encouraging self-reflection. |
| The self-monitoring interface within the digital psychoeducation platform was identified as a key motivator for consistent use. Some participants reported changes in adherence patterns, noting higher levels of adherence during the initial phase of engagement. Furthermore, installing the application on personal mobile devices appeared to enhance motivation and encourage continued use at home. However, certain participants bypassed the intended daily progression of the intervention and accessed later content prematurely, either due to a lack of instruction or misunderstanding of the program’s structure. This led some individuals to appear more advanced during face-to-face therapeutic sessions than their actual progress suggested, potentially confounding clinicians’ evaluations. Overall, the self-monitoring log interface was seen as instrumental in fostering habitual usage and supporting behavioral consistency. While mobile accessibility increased engagement, the absence of guidance in some cases resulted in unintended usage patterns that could distort perceived treatment progress during clinical evaluations. |
| **Attitudes and experiences toward the digital psychoeducation** |
| **Attitude Towards the Approach** |
| Upon initial exposure to the intervention, most participants expressed curiosity and interest in the novelty of the digital psychoeducation approach, noting that it differed significantly from traditional treatment models and effectively stimulated their motivation to engage. Particularly noteworthy was the deep cultural embedding of the content: the narrative scenarios were closely aligned with the lived experiences of local Indigenous populations, which contributed to the intervention’s high level of acceptability and perceived cultural relevance. This culturally sensitive design enhanced both the ecological validity and the overall user receptiveness to the program. |
| However, some participants reported that when the system delivered feedback on incorrect choices, the tone was occasionally perceived as overly direct or judgmental. Such feedback could provoke negative emotional reactions, including anxiety, frustration, or self-doubt. These negative emotions were seen to reduce users' sense of self-efficacy and, in some cases, diminished their willingness to continue engaging with the intervention, thereby affecting their overall perception of the program. A few participants exhibited withdrawal or avoidance behaviors in response to failure-related feedback, highlighting the need for a more balanced feedback mechanism that combines error signaling with psychological support in order to maintain user motivation and emotional resilience. Overall, participants' initial attitudes toward the intervention were shaped by both the cultural resonance of the content and the quality of the feedback experience, which in turn influenced their behavioral persistence and program acceptance. |
| **Attitude Towards the Components** |
| Participants generally reported positive attitudes toward the various components of the digital psychoeducation program. In particular, the narrative-based situational tasks were frequently cited as emotionally resonant. Several respondents mentioned that specific tasks—such as taking medication on time or returning home for ancestral rituals—mirrored their own life experiences, enhancing the personal relevance of the scenarios and increasing emotional engagement and acceptance of the intervention.  Nonetheless, some participants noted a misalignment between certain program content and their own cultural background or daily practices, suggesting that the intervention could benefit from further improvements in cultural inclusivity and representational diversity. This includes better adaptation to variations in gender, social roles, and lived experiences.  In addition to cultural relevance, feedback also varied regarding the difficulty of tasks, reflecting individual differences in cognitive resources and operational capacities. These responses indicate the importance of considering accessibility across populations with varying physical and cognitive abilities. Specific inclusive strategies could include adjustable difficulty levels, customizable user interfaces, and assistive features such as subtitles and audio narration. Such design elements can foster equitable access and optimize user experiences across diverse participant groups. Overall, participant attitudes toward digital psychoeducation modules were strongly influenced by both cultural relevance and functional adaptability.  **Engagement**  Most interviewees reported experiencing a high degree of immersion during their use of the digital psychoeducation platform, gradually perceiving the virtual characters as trustworthy companions. This immersive experience contributed to the development of emotional bonds with the media characters, thereby enhancing users’ engagement and sustaining their participation in the intervention.  For individuals with schizophrenia—who often face social exclusion and stigma in real-life contexts—such carefully designed virtual characters, and interactive scenarios offered a bias-free and non-discriminatory social environment. This environment helped reduce feelings of isolation and strengthened their sense of social connectedness, potentially improving their ability to reintegrate into society.  By simulating everyday interactions and community-based scenarios, the intervention enabled participants to safely practice making decisions and behavioral choices in realistic yet controlled settings. Additionally, these virtual experiences facilitated the acquisition of basic social and life skills, which are transferable to real-life situations and may support participants in addressing social and functional recovery challenges more effectively. Therefore, immersion and character attachment not only contributed to increased engagement but also played a supplementary role in supporting the rehabilitation of social functioning among individuals with serious mental illness. |
| **Expectations of the digital psychoeducation** |
| **Digital Psychoeducation Content** |
| The continuous updating of digital psychoeducation content plays a critical role in sustaining user motivation and long-term intervention engagement. In the initial stages, the novel narrative structure and interactive experience elicited strong interest and participation motivation among users. However, as the storyline progressed and participants were repeatedly exposed to the same elements, some began to report fatigue and diminishing interest, with a few even discontinuing the intervention altogether. This phenomenon highlights the necessity of maintaining content dynamism and a moderate level of complexity to support long-term engagement. |
| **Digital Psychoeducation Function** |
| Regarding functional design, participants expressed a strong demand for reminder and review features. Informed by self-regulation theory, the reminder function was widely perceived as a key mechanism for improving adherence and time management, particularly among individuals with cognitive impairments. Systematic notifications helped users identify important events or task milestones, thereby reducing the likelihood of intervention dropout due to forgetfulness or attentional lapses. |
